# Evaluation of the Root Canal Centering Ratio and Canal Transportation Associated With Three Rotary File Systems Using Cone Beam Computed Tomography (CBCT) Analysis

**DOI:** 10.7759/cureus.71117

**Published:** 2024-10-08

**Authors:** Srikumar G. P. V., Vaishali Shukla, Megha Ghosh, Sakshi Barde, Mohammed Mustafa, Ahmed A Almokhatieb

**Affiliations:** 1 Department of Conservative Dentistry and Endodontics, Triveni Institute of Dental Sciences, Hospital and Research Centre, Bilaspur, IND; 2 Department of Conservative Dental Sciences, College of Dentistry, Prince Sattam Bin Abdulaziz University, Al-Kharj, SAU; 3 Department of Conservative Dentistry and Endodontics, Centre for Transdisciplinary Research, Saveetha Dental College and Hospitals, Saveetha Institute of Medical and Technical Sciences, Saveetha University, Chennai, IND

**Keywords:** canal transportation, centering ratio, cone beam computed tomography, proglider, protaper gold, protaper next, protaper universal

## Abstract

Aim

The aim of the present study was to evaluate the in vitro comparative assessment of root canal centering ratio and canal transportation associated with ProTaper Universal (PTU) (Dentsply Maillefer, Ballaigues, Switzerland), ProTaper Next (PTN) (Dentsply Maillefer, Ballaigues, Switzerland), and ProTaper Gold (PTG) (Dentsply Tulsa Dental Specialties, Tulsa, OK, USA) rotary file systems, with or without glide-path preparation, using cone beam computed tomography (CBCT) analysis.

Materials and methods

A total of 120 mesial roots of extracted human mandibular first molar teeth were collected and randomly divided into three groups (n = 40) depending on the type of rotary file system used for mesiobuccal root canal instrumentation: Group 1: PTU, Group 2: PTN, and Group 3: PTG rotary file systems. Each group was further divided into two Sub-groups (a and b) with 20 specimens, depending on whether glide-path preparation was performed using the ProGlider (PG) file (Dentsply Maillefer, Ballaigues, Switzerland). Before the root canal instrumentation, mesiobuccal root canals of all specimens were first scanned using the NewTom Go CBCT machine (Cefla S.C., Imola, Italy), and all root canals were then instrumented according to their groups and sub-groups. All rotary files were used according to their manufacturer’s guidelines. Post-instrumentation, CBCT images of all specimens were taken with the same exposure parameters as those used in pre-instrumentation CBCT imaging. The distance between the external root surface and the internal canal wall was measured in both bucco-lingual and mesio-distal planes at 3 mm, 5 mm, and 7 mm levels of the mesiobuccal root canal of all specimens, comparing the pre-instrumentation and post-instrumentation CBCT scans for the evaluation of canal centering ratio and canal transportation using NewTom NNT software (Cefla S.C., Imola, Italy). Data were analyzed using one-way analysis of variance (ANOVA), multiple pairwise Tukey post-hoc tests, and Student’s t-tests; a p-value ≤ 0.05 was considered statistically significant.

Results

Canal centering ratio was significant bucco-lingually and mesio-distally at 3 mm and 5 mm levels between Groups 1a, 1b, and 3a, 3b (p < 0.05). However, at 7 mm bucco-lingually, a significant difference was observed between groups 3a and 3b, and mesio-distally between Groups 2a and 2b (p < 0.05). Canal transportation was significantly bucco-lingually at 3 mm, 5 mm, and 7 mm levels between Groups 3a and 3b (p < 0.05). However, mesio-distally, no statistically significant difference (p > 0.05) was seen between the groups at all three levels of the root canal.

Conclusion

The use of the PG/PTN rotary file system showed the maximum canal centering ratio at all three levels of the root canal compared to the PTU and PTG rotary file systems, whether used with or without a glide-path. The PG/PTN rotary file system showed the least canal transportation at the 3 mm level, while at the 5 mm and 7 mm levels, the PG/PTU rotary file system showed the least canal transportation.

## Introduction

The success of root canal treatment depends on effective root canal debridement by eliminating debris and microorganisms, as well as the ability to keep endodontic files well-centered in the root canal space during instrumentation. Delivering accurate enlargement without unnecessarily weakening the root structure is crucial. A prepared root canal should have a continuously tapered funnel shape while maintaining the original outline form [1]. In curved root canals, instrumentation becomes more difficult due to the tendency of endodontic files to divert away from the original axis of the root canal. Historically, root canal instrumentation involved the use of stainless steel hand files, which frequently resulted in undesirable canal aberrations such as elbows, zips, ledges, and canal transportation [2].

Gambill et al. [3] defined the root canal centering ratio as the measurement of the ability of the file to stay centered in the canal during instrumentation. Canal transportation is defined as the removal of canal wall structure on the outside curve in the apical half of the canal due to the tendency of endodontic files to restore themselves to their original linear shape during canal preparation, which may lead to ledge formation and possible perforation [4]. Canal transportation has two components: direction and deviation. Direction refers to the excessive removal of dentin in a single direction along the axis of the root canal. Deviation is any undesirable departure of the file from the original canal path, which is the distance in millimeters between the pre-instrumentation and post-instrumented root canal [5].

Walia et al. [6] suggested a greater modification in endodontic instruments by replacing stainless steel files with nickel-titanium (Ni-Ti) alloy files. The Ni-Ti files are super-elastic, flex more than stainless steel files, and maintain the original root canal curvature better than stainless steel files during canal instrumentation. ProTaper Gold (PTG) (Dentsply Tulsa Dental Specialties, Tulsa, OK, USA) is a Ni-Ti rotary file system developed with proprietary advanced metallurgy through heat treatment technology. It features the same geometries as the ProTaper Universal (PTU) rotary file system (Dentsply Maillefer, Ballaigues, Switzerland). PTG files exhibit a convex triangular cross-section and progressive taper. These files are available in eight sizes: SX (tip size 19 with a taper of 0.04), S1 (tip size 18 with a taper of 0.02), S2 (tip size 20 with a taper of 0.04), F1 (tip size 20 with a taper of 0.07), F2 (tip size 25 with a taper of 0.08), F3 (tip size 30 with a taper of 0.09), F4 (tip size 40 with a taper of 0.06), and F5 (tip size 50 with a taper of 0.05) [7]. The ProTaper Next (PTN) rotary file system (Dentsply Maillefer, Ballaigues, Switzerland) is made of M-wire and consists of five files: X1 (tip size 17 and taper of 4%), X2 (tip size 25 and taper of 6%), X3 (tip size 30 and taper of 7%), X4 (tip size 40 and taper of 6%), and X5 (tip size 50 and taper of 6%) [8].

West [9] defined a glide path as an initial smooth, radicular tunnel-shaped preparation of a root canal from the canal orifice to the physiologic terminus or apical constriction. The glide path allows all subsequent files to progress smoothly from the coronal orifice, unimpeded, to the apical constriction of root canals. ProGlider (PG) (Dentsply Maillefer, Ballaigues, Switzerland) is a rotary glide-path file manufactured using M-wire Ni-Ti alloy to enhance its flexibility, with a variable progressive taper. PG files have a tip size of 16 and a 2% taper [10].

Various methods have been used for the assessment of canal transportation and root canal centering ratio: radiographic analysis (radiographs provide only two-dimensional images of a three-dimensional object), serial sectioning technique, diafanization, stereomicroscopy, and scanning electron microscopy. However, these methods were found to be invasive in nature, and accurate repositioning of pre- and post-instrumented specimens is highly difficult, with the disadvantage of potential loss of specimens [5]. Cone beam computed tomography (CBCT) utilizes a cone-shaped X-ray beam and an area detector that captures a cylindrical volume of data in one acquisition. It is a non-invasive diagnostic method with excellent reproducibility, providing several images of a single root canal at various levels and in different planes, used for accurate analysis of root canal centering ratio and canal transportation [5].

The aim of the present study was to evaluate the root canal centering ratio and canal transportation associated with PTU, PTN, and PTG rotary file systems, with or without glide-path preparation, using CBCT analysis. This hypothesis will guide the investigation into how different rotary file systems and glide-path preparation techniques affect the outcomes of root canal instrumentation, as measured by CBCT analysis.

## Materials and methods

An in-vitro study was conducted in the Department of Conservative Dentistry and Endodontics after obtaining the Institutional Ethical Committee clearance certificate, TIDSHRC/IEC/2021/D007, from the Triveni Institute of Dental Sciences, Hospital and Research Centre, Bilaspur, India. The study sample consisted of 120 freshly extracted human permanent mandibular first molar teeth, following strict inclusion criteria. The inclusion criteria included all teeth that were examined under a stereomicroscope (Olympus SZ61; Olympus Optical Co., Tokyo, Japan) at 10x magnification to ensure that they were intact, devoid of clinically detectable fractures or cracks, and had open root apices. Digital periapical radiographs of the teeth were taken using radiovisiography (RVG) (Kodak; Carestream Health India Pvt Ltd, Maharashtra, India) in buccolingual and mesiodistal directions to confirm that the collected specimens had not been previously endodontically treated, were devoid of resorptive defects and calcifications, and only included teeth with two separate mesial root canals and apical foramina, with an angle of curvature of the mesiobuccal root canal between 20° and 35°, as assessed following Schneider’s criteria [11]. Furthermore, specimens that did not meet the operational criteria for standardized access cavity preparation or instrumentation procedures were excluded.

All specimens were used within one month of extraction. The collected teeth were cleaned of superficial debris, calculus, and residual tissue tags using ultrasonic instruments. Occupational Safety and Health Administration (OSHA), Centers for Disease Control and Prevention (CDC), and other recommendations and guidelines were strictly followed during the collection, sterilization, and handling of extracted teeth to prevent any biohazard transmission. All teeth were then stored in 0.5% thymol at room temperature until use.

Endodontic access cavity preparation was done using an Endo access bur no. 2 (Dentsply Maillefer, Ballaigues, Switzerland) attached to a high-speed contra-angled air rotor handpiece (NSK Ltd., Tokyo, Japan) to gain access only to the mesiobuccal root canals of all teeth. A no. 10 K-file (Mani, Inc., Tochigi, Japan) was placed into the root canals, with the tip of the trial file just visible at the apical foramen, and the working length was determined by subtracting 0.5 mm from this length, thus establishing the final working length. All teeth were then hemisected using a diamond disc (DFS, Langen, Germany) attached to a micromotor straight handpiece at low speed, under water coolant, into mesial and distal halves at the level of the furcation; the distal halves were discarded. The mesial halves of the teeth were not decoronated and were numbered 1 to 120, with markings made on the teeth for ease of identification of the mesiobuccal root canal orifices using a permanent marker pen (Kokuyo Camlin Pvt. Ltd., Mumbai, India). All specimens were then randomly divided into three groups, with 40 specimens each, depending on the type of rotary file system used for mesiobuccal root canal instrumentation: Group 1 (PTU), Group 2 (PTN), and Group 3 (PTG). Each group was further divided into two Sub-groups (a and b), with 20 specimens each, depending on whether glide-path preparation was performed using a PG file.

In Sub-group 1a (n = 20), PTU rotary files were used for root canal instrumentation in a crown-down technique at 250 RPM (revolutions per minute) and 2.8 Ncm torque until the full working length was reached in the sequence of SX, S1, S2, F1, F2, and F3 files, without the use of the PG rotary file for initial glide-path preparation. In Sub-group 1b (n = 20), initial glide-path preparation of root canals was done using the PG rotary file up to the full working length at a speed of 300 RPM and 2-5.2 Ncm torque with light apical pressure, followed by instrumentation of the root canals with PTU rotary files sequentially up to size F3, following manufacturer instructions.

In Sub-group 2a (n = 20), PTN rotary files were used for root canal instrumentation in a crown-down technique at 300 RPM and 2-5.2 Ncm torque until the full working length was reached in the sequence of X1, X2, and X3 files, without the use of the PG rotary file for initial glide-path preparation. In Sub-group 2b (n = 20), initial glide-path preparation of root canals was done using a PG rotary file, followed by instrumentation of the root canals with PTN rotary files sequentially up to size X3, following manufacturer instructions.

In Sub-group 3a (n = 20), PTG rotary files were used for root canal instrumentation in a crown-down technique at 250 RPM and 2.8 Ncm torque until the full working length was reached in the sequence of SX, S1, S2, F1, F2, and F3 files, without the use of the PG rotary file for initial glide-path preparation. In Sub-group 3b (n = 20), initial glide-path preparation of root canals was done using a PG rotary file, followed by instrumentation of the root canals with PTG rotary files sequentially up to size F3, following manufacturer instructions.

All rotary file systems were operated while attached to a torque-controlled endomotor handpiece (CanalPro CL2, Coltene Endo; Coltene Whaledent Pvt. Ltd., Alstatten, Switzerland), following manufacturer instructions. Each rotary file (PTU, PTN, PTG, and PG) was used for the instrumentation of five root canals and then discarded, followed by the use of new files. In each root canal, during instrumentation, 2 mL of 17% EDTA (ethylenediaminetetraacetic acid) solution (Prevest Denpro, Jammu, India), 2 mL of 3% sodium hypochlorite solution (Neelkaanth Health Care Pvt. Ltd., Ahmedabad, India), and 2 mL of distilled water (Sadbhavna Chemicals, Morbi, India) were used as root canal irrigants in a disposable syringe with Max-I-Probe (Dentsply Maillefer, Ballaigues, Switzerland) irrigation needles. To prevent any inter-operator variability, all specimens were instrumented by a single endodontist.

All specimens were then embedded up to the level of the cervical line in a template of self-cure acrylic resin blocks (DPI RR Cold Cure; Dental Product of India, Mumbai, India) simulated in the mandibular arch form to obtain a stable, fixed position during their exposure to CBCT imaging and also for ease of handling during root canal instrumentation [5]. The template was then horizontally fitted to the chin support of the CBCT machine, with its occlusal plane parallel to the plate of the CBCT machine and the roots of the teeth aligned perpendicularly to the X-ray beam.

**Figure 1:**
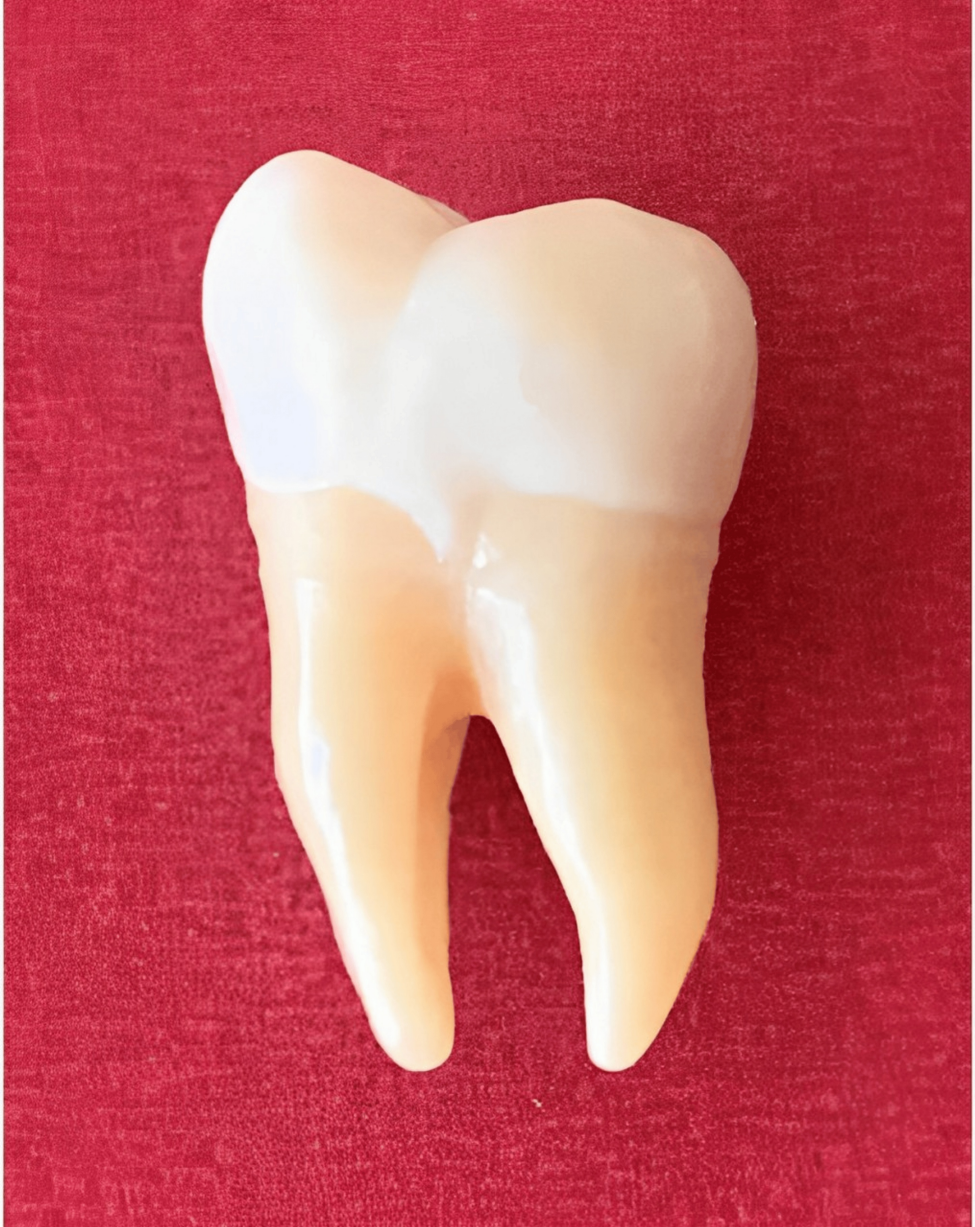
Extracted molar specimen for the study.

Prior to the root canal instrumentation, mesio-buccal root canals of all specimens were first scanned using the NewTom Go CBCT machine (Cefla S.C., Imola, Italy) with the exposure settings of 90 kVp, 10 mA, and 16.8 seconds for high resolution, a field of view (FOV) of 6 × 6 cm, and a voxel size of 160-80 μm. Both bucco-lingual and mesio-distal dimensions of the root canals were recorded at 3 mm, 5 mm, and 7 mm from the pre-determined working length, and the obtained pre-instrumentation CBCT images were stored on the computer’s hard disk for further comparison with the corresponding post-instrumentation CBCT images (Figure 1).

**Figure 2:**
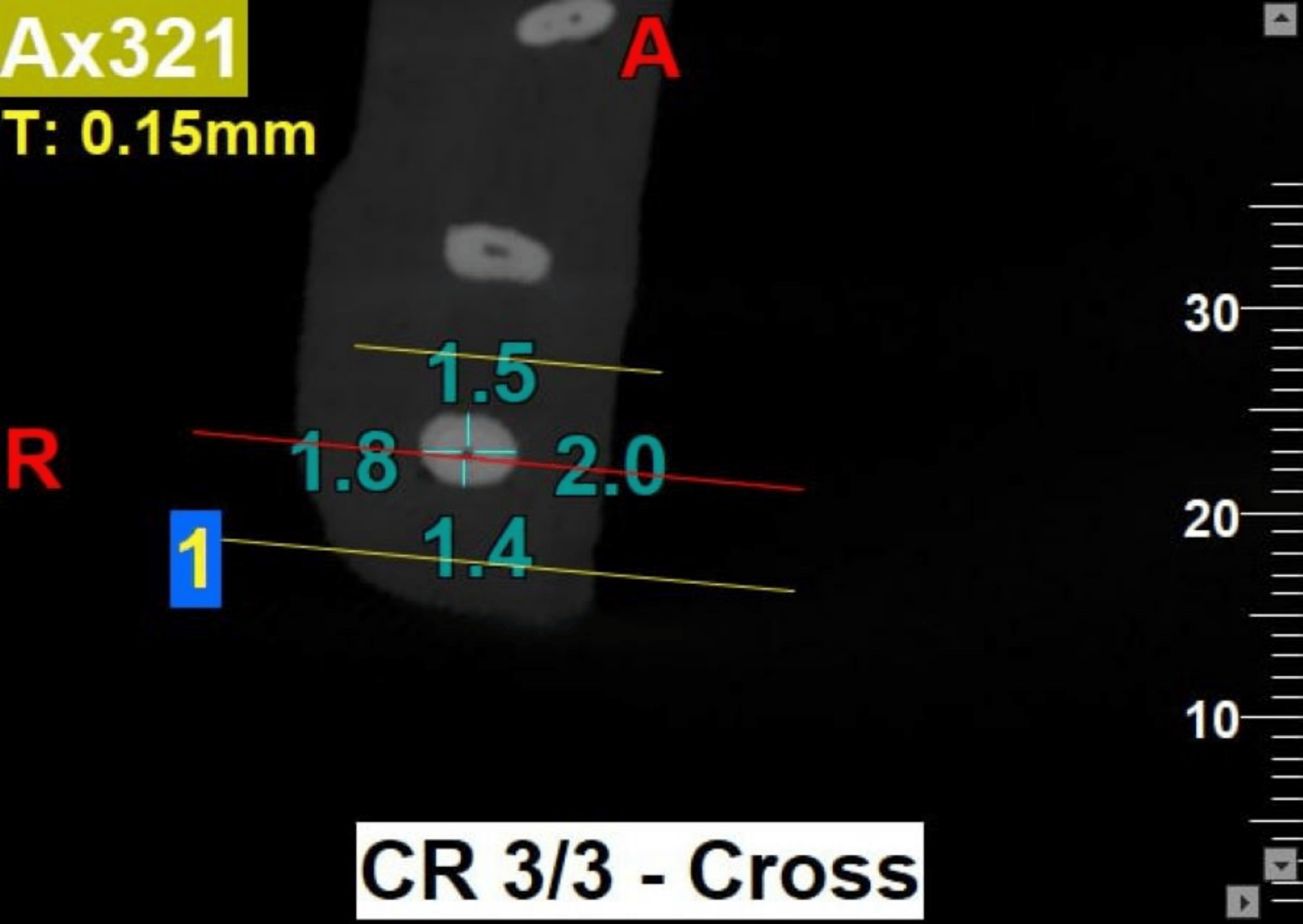
Pre-instrumentation CBCT at 3 mm from pre-determined working length. CBCT: Cone beam computed tomography

**Figure 3:**
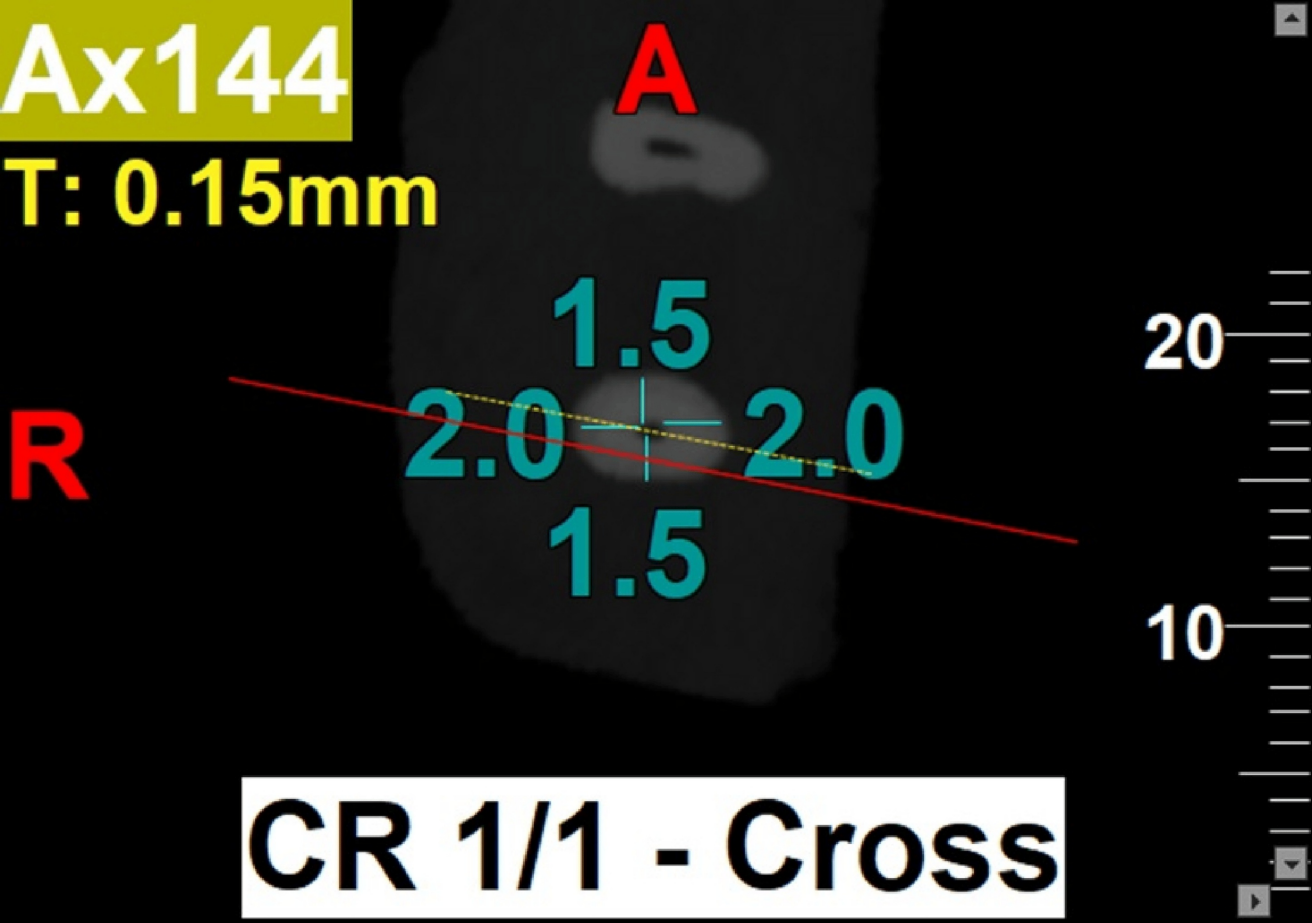
Post-instrumentation CBCT at 3 mm from pre-determined working length. CBCT: Cone beam computed tomography

Post-instrumentation of all specimens, CBCT imaging was repeated with the same exposure parameters as in pre-instrumentation CBCT imaging (Figures 2-3).

The distance between the external root surface and the internal canal wall was measured in both bucco-lingual and mesio-distal planes at 3 mm, 5 mm, and 7 mm levels of mesio-buccal root canal from the pre-determined working length of all specimens for the evaluation of root canal centering ratio and canal transportation between the pre-instrumentation and post-instrumentation CBCT images using NewTom NNT software (Cefla S.C, Imola, Italy) with the help of the formula proposed by Gambill et al. [3] and the data was recorded.

The root canal centering ratio is calculated for both the mesio-distal and bucco-lingual planes. For the mesio-distal plane, the ratio is given by (m1 - m2)/(d1 - d2), while for the bucco-lingual plane, it is expressed as (b1 - b2)/(l1 - l2). Similarly, the degree of canal transportation is assessed using the same planes: for the mesio-distal plane, it is calculated as (m1 - m2)/(d1 - d2), and for the bucco-lingual plane, it is represented by (b1 - b2)/(l1 - l2).

Here, m1 is the shortest distance between mesial borders of the root canal before instrumentation and m2 is the shortest distance between mesial borders of the root canal after instrumentation. d1 is the shortest distance between the distal borders of the root canal before instrumentation and d2 is the shortest distance between distal borders of the root canal after instrumentation. b1 is the shortest distance between the buccal borders of the root canal before instrumentation and b2 is the shortest distance between the buccal borders of the root canal after instrumentation, l1 is the shortest distance between the lingual borders of the root canal before instrumentation and l2 is the shortest distance between the lingual borders of the root canal after instrumentation [3].

Evaluation of root canal centering ratio

The larger value was taken as the denominator. A value of ‘1’ indicates optimal root canal centering ability, whereas any value other than ‘1’ indicates changes in the root canal pathway away from the central axis. The closer the obtained value is to ‘1’, the higher the centering ratio; conversely, the closer the value is to ‘0’, the lower the centering ratio. The obtained data was then recorded and tabulated.

Evaluation of canal transportation

A value of ‘0’ means no canal transportation. The closer the obtained value is to ‘0’, the less canal transportation occurs. Positive values indicate mesial or buccal canal transportation, while negative values indicate distal or lingual canal transportation. The obtained data was then recorded and tabulated. To prevent any inter-observer variability, all CBCT images were evaluated by a single dentomaxillofacial radiologist.

Statistical analysis

IBM SPSS Statistics for Windows, Version 24 (Released 2016; IBM Corp., Armonk, NY, USA), was used for data analysis. The mean and standard deviation (SD) values in millimeters (mm) of canal centering ratio and canal transportation were obtained for the three groups. One-way analysis of variance (ANOVA) was used for intra-group comparison to determine any statistically significant differences among them. A p-value of ≤0.05 was considered statistically significant. Multiple pair-wise inter-group comparisons among the three groups were conducted using the Tukey post-hoc test. A comparison of canal centering ability and canal transportation between the Sub-groups (1a and 1b, 2a and 2b, 3a and 3b), with and without glide-path preparation, was performed using the Student’s t-test; a p-value of ≤0.05 was considered statistically significant.

## Results

The highest canal centering ratio was seen at the 3 mm level in Group 2b (PG/PTN) file systems, in both bucco-lingual and mesio-distal planes, with means of 0.68 mm and 0.56 mm, respectively. At the 5 mm level, Group 3b (PG/PTG), followed by Group 2b (PG/PTN) file systems, showed the highest canal centering ratio in both bucco-lingual and mesio-distal planes, with means of 0.60 mm and 0.54 mm, and 0.61 mm and 0.53 mm, respectively. At the 7 mm level, in the bucco-lingual plane, Group 1a (PTU), followed by Group 2b (PG/PTN) file systems, showed the highest canal centering ratio, with means of 0.59 mm and 0.57 mm, respectively. In the mesio-distal plane, Group 2b (PG/PTN), followed by Group 1a (PTU) file systems, showed the highest canal centering ratio, with means of 0.65 mm and 0.62 mm, respectively.

The least canal centering ratio was seen in the bucco-lingual plane, at the 3 mm level in Group 3b (mean 0.17 mm), at the 5 mm level in Group 1a, followed by Group 3a, with means of 0.24 mm and 0.26 mm, respectively, and at the 7 mm level in Group 3a (mean 0.37 mm). In the mesio-distal plane, at the 3 mm level, Group 1a (mean 0.21 mm) followed by Group 2a (mean 0.24 mm) and Group 3b (mean 0.22 mm) showed the least centering ratio. At the 5 mm level, Group 1b (mean 0.36 mm) and at the 7 mm level, Group 2a (mean 0.34 mm), followed by Group 3a (mean 0.38 mm), showed the least centering ratio, as shown in Tables 1-2.

**Table 1:** One way ANOVA and Tukey post-hoc tests for comparison of canal centering ratio in bucco-lingual plane at 3 mm, 5 mm, and 7 mm levels of root canal. P: Probability; SD: Standard deviation; HS: Highly significant; S: Significant; NS: Not significant; mm: Millimeter; ANOVA: Analysis of variance

Bucco-lingual plane	Groups	No. of specimens	Mean in mm	±SD in mm	p-value	Group-wise comparison (Tukey post-hoc test)		
						1a vs 2a	1a vs 3a	2a vs 3a
At 3-mm level	Group 1a	20	0.23	0.28	0.004; HS	p < 0.01; S	p > 0.05; NS	p > 0.05; NS
	Group 2a	20	0.49	0.36				
	Group 3a	20	0.38	0.4				
						1b vs 2b	1b vs 3b	2b vs 3b
	Group 1b	20	0.47	0.4	0.02; S	p > 0.05; NS	p > 0.05; S	p < 0.05; S
	Group 2b	20	0.68	0.33				
	Group 3b	20	0.17	0.26				
At 5-mm level	Group 1a	20	0.24	0.27	0.23; NS	p > 0.05; NS	p > 0.05; NS	p > 0.05; NS
	Group 2a	20	0.39	0.33				
	Group 3a	20	0.26	0.31				
						1b vs 2b	1b vs 3b	2b vs 3b
	Group 1b	20	0.43	0.38	0.28; NS	p > 0.05; NS	p > 0.05; NS	p > 0.05; NS
	Group 2b	20	0.54	0.35				
	Group 3b	20	0.60	0.27				
At 7-mm level	Group 1a	20	0.59	0.29	0.09; NS	p > 0.05; NS	p > 0.05; NS	p > 0.05; NS
	Group 2a	20	0.55	0.36				
	Group 3a	20	0.37	0.32				
						1b vs 2b	1b vs 3b	2b vs 3b
	Group 1b	20	0.54	0.41	0.93; NS	p > 0.05; NS	p > 0.05; NS	p > 0.05; NS
	Group 2b	20	0.57	0.23				
	Group 3b	20	0.58	0.26				

**Table 2:** One way ANOVA and Tukey post-hoc tests for comparison of canal centering ratio in mesio-distal plane at 3 mm, 5 mm, and 7 mm levels of root canal. P: Probability; SD: Standard deviation; HS: Highly significant, S: Significant, NS: Not significant, mm: millimeter; ANOVA: Analysis of variance

Mesio-distal plane	Groups	No. of specimens	Mean in mm	±SD in mm	p-value	Group-wise comparison (Tukey post-hoc test)		
						1a vs 2a	1a vs 3a	2a vs 3a
At 3-mm level	Group 1a	20	0.21	0.28	0.18; NS	p > 0.05; NS	p > 0.05; NS	p > 0.05; NS
	Group 2a	20	0.24	0.33				
	Group 3a	20	0.36	0.35				
						1b vs 2b	1b vs 3b	2b vs 3b
	Group 1b	20	0.37	0.38	0.01; S	p > 0.05; NS	p > 0.05; NS	p < 0.05; S
	Group 2b	20	0.56	0.41				
	Group 3b	20	0.22	0.22				
At 5-mm level	Group 1a	20	0.52	0.30	0.78; NS	p > 0.05; NS	p > 0.05; NS	p > 0.05; NS
	Group 2a	20	0.45	0.35				
	Group 3a	20	0.48	0.36				
						1b vs 2b	1b vs 3b	2b vs 3b
	Group 1b	20	0.36	0.36	0.02; S	p > 0.05; NS	p < 0.05; S	p > 0.05; NS
	Group 2b	20	0.53	0.42				
	Group 3b	20	0.61	0.28				
At 7-mm level	Group 1a	20	0.62	0.24	0.005; HS	p < 0.05; S	p < 0.05; S	p > 0.05; NS
	Group 2a	20	0.34	0.43				
	Group 3a	20	0.38	0.21				
						1b vs 2b	1b vs 3b	2b vs 3b
	Group 1b	20	0.58	0.35	0.04; S	p > 0.05; NS	p > 0.05; NS	p < 0.05; S
	Group 2b	20	0.65	0.25				
	Group 3b	20	0.39	0.27				

Sub-group comparisons in the bucco-lingual plane of root canals showed that, at the 3 mm level, Group 1b (mean 0.47) had a better centering ratio compared to Group 1a (mean 0.23), with p = 0.034 (significant difference observed). At the 5 mm level, Group 3b (mean 0.60) showed a better centering ratio compared to Group 3a (mean 0.26), with p = 0.006 (highly significant difference observed). At the 7 mm level, Group 3b (mean 0.58) had a better centering ratio compared to Group 3a (mean 0.37), with p = 0.028 (significant difference observed), as shown in Table 3.

**Table 3:** Student's t-test: Comparison of canal centering ratio between the sub-groups in bucco-lingual plane at three levels of root canal. P: Probability; SD: Standard deviation; HS: Highly significant; S: Significant; NS: Not significant; mm: Millimeter

Bucco-lingual plane	Comparison of sub-groups	No. of specimens	Mean in mm	±SD in mm	p-value
					Student's t-test
At 3-mm level	Group 1a	20	0.23	0.28	0.034; S
	Group 1b	20	0.47	0.4	
	Group 2a	20	0.49	0.36	0.24; NS
	Group 2b	20	0.68	0.33	
	Group 3a	20	0.38	0.4	0.14; NS
	Group 3b	20	0.17	0.26	
At 5-mm level	Group 1a	20	0.24	0.27	0.07; NS
	Group 1b	20	0.43	0.38	
	Group 2a	20	0.39	0.33	0.17; NS
	Group 2b	20	0.54	0.35	
	Group 3a	20	0.26	0.31	0.006; HS
	Group 3b	20	0.6	0.27	
At 7-mm level	Group 1a	20	0.59	0.29	0.66; NS
	Group 1b	20	0.54	0.41	
	Group 2a	20	0.55	0.36	0.83; NS
	Group 2b	20	0.57	0.23	
	Group 3a	20	0.37	0.32	0.028; S
	Group 3b	20	0.58	0.26	

Sub-group comparisons in the mesio-distal plane of root canals showed that, at the 3 mm level, Group 1b (mean 0.37) had a better-centering ratio than Group 1a (mean 0.21), with p = 0.065 (significant difference observed), and Group 2b (mean 0.56) showed a better-centering ratio than Group 2a (mean 0.24), with p = 0.009 (highly significant difference observed). At the 5 mm level, Group 3b (mean 0.61) had a better centering ratio than Group 3a (mean 0.48), with p = 0.057 (significant difference observed). At the 7 mm level, Group 2b (mean 0.65) showed a better centering ratio than Group 2a (mean 0.34), with p = 0.02 (significant difference observed), as shown in Table 4.

**Table 4:** Student's t-test: Comparison of canal centering ratio between the sub-groups in mesio-distal plane at three levels of root canal. P: Probability; SD: Standard deviation; HS: Highly significant; S: Significant; NS: Not significant; mm: Millimeter

Mesio-distal plane	Comparison of sub-groups	No. of specimens	Mean in mm	±SD in mm	p-value
					Student's t-test
At 3-mm level	Group 1a	20	0.21	0.28	0.065; S
	Group 1b	20	0.37	0.38	
	Group 2a	20	0.24	0.33	0.009; HS
	Group 2b	20	0.56	0.41	
	Group 3a	20	0.36	0.35	0.14; NS
	Group 3b	20	0.22	0.22	
At 5-mm level	Group 1a	20	0.52	0.3	0.13; NS
	Group 1b	20	0.36	0.36	
	Group 2a	20	0.45	0.35	0.51; NS
	Group 2b	20	0.53	0.42	
	Group 3a	20	0.48	0.36	0.057; S
	Group 3b	20	0.61	0.28	
At 7-mm level	Group 1a	20	0.62	0.24	0.46; NS
	Group 1b	20	0.58	0.35	
	Group 2a	20	0.34	0.43	0.02; S
	Group 2b	20	0.65	0.25	
	Group 3a	20	0.38	0.21	0.89; NS
	Group 3b	20	0.39	0.27	

Root canal transportation in the bucco-lingual plane showed that, at the 3 mm level, the use of PG/PTN files resulted in the least canal transportation, with a mean of -0.04 mm. At the 5 mm level, the use of PG/PTU files showed no canal transportation, with a mean of 0 mm. At the 7 mm level, the use of PG/PTU files again showed the least canal transportation, with a mean of -0.03 mm, as shown in Table 5.

**Table 5:** One way ANOVA and Tukey post-hoc tests for comparison of canal transportation in bucco-lingual plane at 3 mm, 5 mm, and 7 mm levels of root canal. P: Probability; SD: Standard deviation; HS: Highly significant; S: Significant; NS: Not significant; mm: Millimeter; ANOVA: Analysis of variance

Bucco-lingual plane	Groups	No. of specimens	Mean in mm	±SD in mm	p-value	Group-wise comparison (Tukey post-hoc test)		
						1a vs 2a	1a vs 3a	2a vs 3a
At 3-mm level	Group 1a	20	-0.14	0.33	0.18; NS	p > 0.05; NS	p > 0.05; NS	p > 0.05; NS
	Group 2a	20	0.1	0.39				
	Group 3a	20	-0.08	0.52				
						1b vs 2b	1b vs 3b	2b vs 3b
	Group 1b	20	0.14	0.45	0.005; HS	p > 0.05; NS	p > 0.05; NS	p < 0.05; S
	Group 2b	20	-0.04	0.25				
	Group 3b	20	0.32	0.25				
At 5 mm level	Group 1a	20	-0.2	1.02	0.43; NS	p > 0.05; NS	p > 0.05; NS	p > 0.05; NS
	Group 2a	20	0.05	0.4				
	Group 3a	20	-0.25	0.72				
						1b vs 2b	1b vs 3b	2b vs 3b
	Group 1b	20	0	1.1	0.49; NS	p > 0.05; NS	p > 0.05; NS	p > 0.05; NS
	Group 2b	20	-0.15	0.33				
	Group 3b	20	0.11	0.29				
At 7-mm level	Group 1a	20	-0.22	0.48	0.009; HS	p > 0.05; NS	p > 0.05; NS	p < 0.05; S
	Group 2a	20	-0.08	0.41				
	Group 3a	20	-0.58	0.62				
						1b vs 2b	1b vs 3b	2b vs 3b
	Group 1b	20	-0.03	0.44	0.17; NS	p > 0.05; NS	p > 0.05; NS	p > 0.05; NS
	Group 2b	20	-0.13	0.32				
	Group 3b	20	-0.26	0.38				

In the mesio-distal plane, at the 3 mm level, the use of both PG/PTU and PG/PTG file systems showed the least canal transportation, with a mean of -0.02 mm. At the 5 mm level, the use of PG/PTU files showed the least canal transportation, with a mean of -0.01 mm. At the 7 mm level, both PTN and PTG files without glide-path showed the least canal transportation, with a mean of -0.08 mm, as shown in Table 6.

**Table 6:** One way ANOVA and Tukey post-hoc tests for comparison of canal transportation in mesio-distal plane at 3 mm, 5 mm, and 7 mm levels of root canal. P: Probability; SD: Standard deviation; HS: Highly significant; S: Significant; NS: Not significant; mm: Millimeter; ANOVA: Analysis of variance

Mesio-distal plane	Groups	No. of specimens	Mean in mm	±SD in mm	p-value	Group-wise comparison (Tukey post-hoc test)		
						1a vs 2a	1a vs 3a	2a vs 3a
At 3-mm level	Group 1a	20	0.05	0.18	0.43; NS	p > 0.05; NS	p > 0.05; NS	p > 0.05; NS
	Group 2a	20	0.05	0.24				
	Group 3a	20	-0.04	0.41				
						1b vs 2b	1b vs 3b	2b vs 3b
	Group 1b	20	-0.02	0.35	0.67; HS	p > 0.05; NS	p > 0.05; NS	p > 0.05; NS
	Group 2b	20	0.08	0.09				
	Group 3b	20	-0.02	0.31				
At 5-mm level	Group 1a	20	0.13	0.39	0.09; NS	p > 0.05; NS	p > 0.05; NS	p > 0.05; NS
	Group 2a	20	0.09	0.27				
	Group 3a	20	-0.06	0.15				
						1b vs 2b	1b vs 3b	2b vs 3b
	Group 1b	20	-0.01	0.21	0.02; S	p > 0.05; NS	p < 0.05; S	p > 0.05; NS
	Group 2b	20	0.13	0.1				
	Group 3b	20	0.15	0.24				
At 7-mm level	Group 1a	20	0	0.15	0.73; NS	p > 0.05; NS	p > 0.05; NS	p > 0.05; NS
	Group 2a	20	-0.08	0.52				
	Group 3a	20	-0.08	0.29				
						1b vs 2b	1b vs 3b	2b vs 3b
	Group 1b	20	0.07	0.19	0.83; NS	p > 0.05; NS	p > 0.05; NS	p > 0.05; NS
	Group 2b	20	0.09	0.06				
	Group 3b	20	0.03	0.41				

Sub-group comparison in the bucco-lingual plane of root canals showed that, at the 3 mm level, Group 2a (mean 0.1 mm) showed the least canal transportation. At the 5 mm level, Group 1b (mean 0) showed no canal transportation. At the 7 mm level, Group 1b (mean -0.03 mm) showed the least canal transportation. A statistically highly significant difference (p < 0.05) was observed in canal transportation between Groups 3a and 3b (p = 0.003) at the 3 mm level. A statistically significant difference was also noted between Groups 3a and 3b at both the 5 mm and 7 mm levels (p = 0.04), as shown in Table 7.

**Table 7:** Student's t-test: Comparison of canal transportation between the sub-groups in bucco-lingual plane at three levels of root canal. P: Probability; SD: Standard deviation; HS: Highly significant; S: Significant; NS: Not significant; mm: Millimeter

Bucco-lingual plane	Comparison of sub-groups	No. of specimens	Mean in mm	±SD in mm	p-value
					Student's t-test
At 3-mm level	Group 1a	20	-0.14	0.33	0.03; S
	Group 1b	20	0.14	0.45	
	Group 2a	20	0.1	0.39	0.18; NS
	Group 2b	20	-0.04	0.25	
	Group 3a	20	-0.08	0.52	0.003; HS
	Group 3b	20	0.32	0.25	
At 5-mm level	Group 1a	20	-0.2	1.02	0.55; NS
	Group 1b	20	0	1.1	
	Group 2a	20	0.05	0.4	0.09; NS
	Group 2b	20	-0.15	0.33	
	Group 3a	20	-0.25	0.72	0.04; S
	Group 3b	20	0.11	0.29	
At 7-mm level	Group 1a	20	-0.22	0.48	0.55; NS
	Group 1b	20	-0.03	0.44	
	Group 2a	20	-0.08	0.41	0.09; NS
	Group 2b	20	-0.13	0.32	
	Group 3a	20	-0.58	0.62	0.04; S
	Group 3b	20	-0.26	0.38	

Sub-group comparison in the mesio-distal plane of root canals showed that, at the 3 mm level, Groups 1b and 3b (mean -0.02 mm) showed the least canal transportation. At the 5 mm level, Group 1b (mean -0.01 mm) also showed the least canal transportation. At the 7 mm level, Group 1a (mean 0) showed no canal transportation. However, no statistically significant difference (p > 0.05) was observed in canal transportation between Groups 1a and 1b, Groups 2a and 2b, and Groups 3a and 3b at all three levels of the root canal, as shown in Table 8.

**Table 8:** Student's t-test: Comparison of canal transportation between the sub-groups in mesio-distal plane at three levels of root canal. P: Probability; SD: Standard deviation; HS: Highly significant; S: Significant; NS: Not significant; mm: Millimeter

Mesio-distal plane	Comparison of sub-groups	No. of specimens	Mean in mm	±SD in mm	p-value
					Student's t-test
At 3-mm level	Group 1a	20	0.05	0.18	0.43; NS
	Group 1b	20	-0.02	0.35	
	Group 2a	20	0.05	0.24	0.60; NS
	Group 2b	20	0.08	0.09	
	Group 3a	20	-0.04	0.41	0.86; NS
	Group 3b	20	-0.02	0.31	
At 5-mm level	Group 1a	20	0.13	0.39	0.43; NS
	Group 1b	20	-0.01	0.21	
	Group 2a	20	0.09	0.27	0.17; NS
	Group 2b	20	0.13	0.1	
	Group 3a	20	-0.06	0.15	0.53; NS
	Group 3b	20	0.15	0.24	
At 7-mm level	Group 1a	20	0	0.15	0.23; NS
	Group 1b	20	0.07	0.19	
	Group 2a	20	-0.08	0.52	0.16; NS
	Group 2b	20	0.09	0.06	
	Group 3a	20	-0.08	0.29	0.19; NS
	Group 3b	20	0.03	0.41	

**Figure 4:**
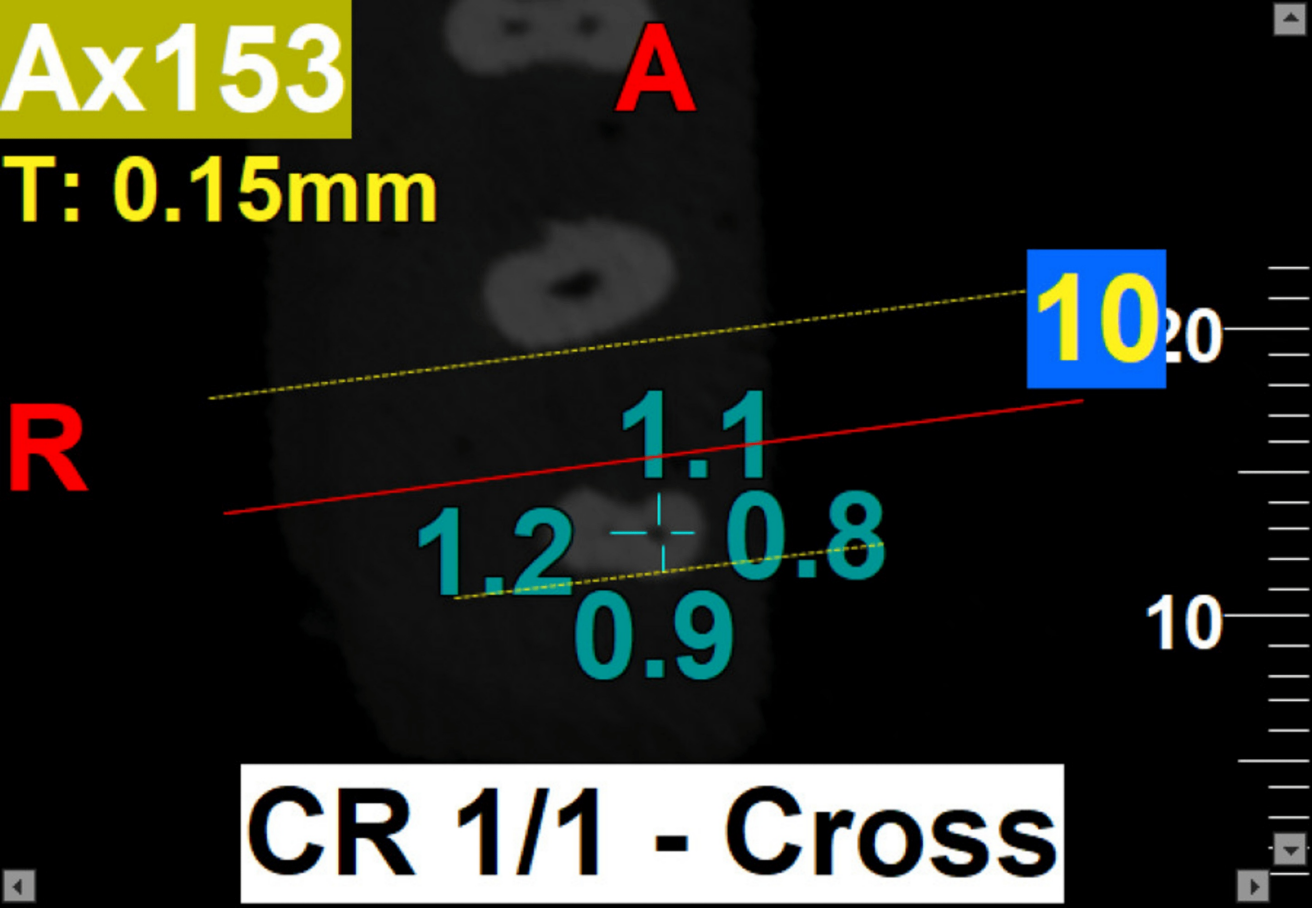
At the 3 mm level, the PG/PTG file system shows the least canal centering ratio. PTG: ProTaper Gold; PG: ProGlider

**Figure 5:**
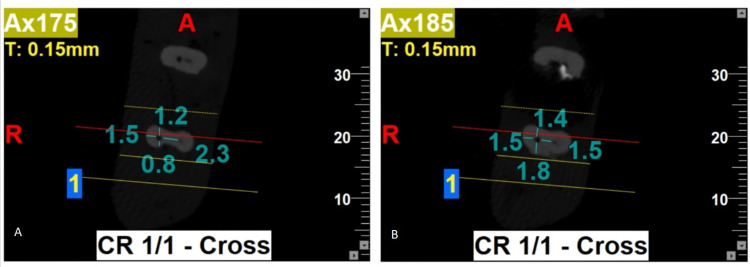
At 5 mm (A) and 7 mm (B) levels, the PTG system shows the least canal centering ratio. PTG: ProTaper Gold

The results of the present study showed that the use of PG rotary file for initial glide-path preparation, followed by the use of PTU, PTN, and PTG rotary file systems for root canal instrumentation, resulted in a better canal centering ratio at three levels (3 mm, 5 mm, and 7 mm) of the root canal in both bucco-lingual and mesio-distal planes. However, the use of PG/PTN rotary files (Group 2b) for canal instrumentation demonstrated the maximum canal centering ratio at all three levels compared to other rotary file systems used either with or without glide-path preparation (Figures 2-3). Conversely, at the 3 mm level, PG/PTG and, at the 5 mm and 7 mm levels, PTG file systems showed the least canal centering ratio (Figures 4-5).

The use of PTN (Group 2a) and PG/PTN (Group 2b) rotary file systems for canal instrumentation showed the least canal transportation at the 3 mm level. In contrast, the PG/PTU (Group 1b) followed by the PTU (Group 1a) rotary file systems exhibited the least canal transportation at the 5 mm and 7 mm levels of the root canal, both in bucco-lingual and mesio-distal planes, compared to the other rotary file systems used either with or without glide-path preparation.

## Discussion

In our study, mesiobuccal root canals of permanent mandibular first molar teeth were selected because they typically show noticeable curvature in the apical and middle thirds and are highly susceptible to various procedural mishaps during root canal instrumentation. Hence, we chose three levels - 3 mm, 5 mm, and 7 mm - representing the apical, middle, and coronal thirds of the root canal for the evaluation of canal centering ratio and canal transportation of three rotary file systems used either with or without initial glide-path preparation using a PG file for root canal instrumentation, as assessed through CBCT analysis. In our study, the crowns corresponding to the mesial roots were kept intact to mimic clinical conditions, where the interference of cervical dentin could create tension on the files during canal instrumentation [12]. The mesiobuccal root canals with an angle of curvature between 20° and 35°, as assessed by Schneider’s criteria [11], were chosen, as this range is considered moderate curvature according to the American Association of Endodontists (AAE) Endodontic Case Difficulty Assessment (ECDA), covering a larger number of cases [7]. No file separation or fracture occurred in our study regarding any of the rotary file systems used.

In our study, the use of the PG rotary file for initial glide-path preparation, followed by the PTN rotary file system for canal instrumentation, showed the highest canal centering ratio at 3 mm, 5 mm, and 7 mm, as well as the least canal transportation at the 3 mm and 7 mm levels of the root canals. This can be attributed to the glide path created using the PG file, which resulted in less stress on the PTN files during instrumentation. Both PTN and PG rotary files are made with innovative M-wire Ni-Ti technology, and the manufacturer suggests that the use of a PG rotary file corresponds to using a size 10 K (Kerr) hand file in the canal [10]. The findings of our study corroborate those of Elnaghy and Elsaka [10], who reported that the use of a smaller size, lesser taper, and flexible PG rotary file for initial glide-path preparation helps preserve the original root canal geometry, thus enhancing root canal instrumentation with the PTN rotary file system without causing any canal aberrations.

The better performance of the PTN rotary file system might also be due to its modified non-cutting tip design, off-centered mass of rotation with a rectangular cross-section, regressive taper enabling a reduced pattern of contact, screwing-in effect, and taper lock of the file with the dentinal walls of the root canal. This design provides a snake-like swaggering motion of the file, better flutes unloading of collected dentin debris, and improved apical progression of the file in the root canal with enhanced flexibility [10]. The use of PTN files after the initial glide-path preparation of root canals with a PG file reduces the possibility of misdirected file instrumentation and subsequent root canal transportation [13]. Tavanafar et al. [14] concluded in their study that the PTN rotary file system showed better canal centering ability and the least canal transportation at the apical thirds of root canals compared to PTU and WaveOne file systems. Zanette et al. [15] reported that initial glide-path preparation of the root canal enhances instrumentation with PTN files, as it creates a pathway to the full working length, thus avoiding excessive binding of files against the dentinal walls of the root canal. The findings of our study also corroborate those of an in-vitro study by Türker and Uzunoğlu [16], which reported better canal centering ability and the least canal transportation with the sequential use of a PG rotary file for initial glide-path preparation, followed by the use of PTN rotary files for complete canal instrumentation. PTN files are also suitable for use either with or without glide-path files without deviating from the original canal geometry.

In our study, it was found that all three rotary file systems, whether used with or without the PG rotary file for initial glide-path preparation, showed greater canal transportation at the apical 3 mm compared to the 5 mm (middle-third) and 7 mm (coronal-third) levels of the root canal. These findings corroborate the results of Ceyhanli et al. [17] and Delgoshayi et al. [18], which showed greater canal transportation at the apical third compared to other levels of the root canal. In our study, initial glide-path preparation with a PG file followed by the use of PTU rotary files also showed the least canal transportation at all three levels of the root canal. These findings are in agreement with the results of Elnaghy and Elsaka [7] and Delgoshayi et al. [18]. The modified cross-sectional design of PTU rotary files, with a rounded safe tip, smaller contact area with the dentin wall, and U-shaped grooves added at the convex triangular sides of the file, improves its flexibility and reduces canal transportation [19].

In our study, PTG rotary files exhibited the least canal centering ratio and the maximum canal transportation, either with or without the use of a PG rotary file for instrumentation. These findings corroborate a study by Singh et al. [20], which reported that PTG files are aggressive cutting files that remove excessive dentin and show the least canal centering ratio and maximum canal transportation. The aggressive cutting ability of PTG files along the dentin walls of the root canal is likely due to their sharp cutting edges, convex triangular core mass, which causes a reduction in file flexibility, increased stiffness of the file tip, and a flute design combining multiple tapers within the shaft, up to 19%, as confirmed by Paqué et al. [21].

Wu et al. [22] reported that apical canal transportation greater than 0.3 mm decreases the quality of the apical seal during root canal filling and could negatively impact the long-term success of root canal treatment. The results of our study showed that none of the instrumented specimens reached the above-mentioned critical level (>0.3 mm) of apical canal transportation (mean values in bucco-lingual and mesio-distal planes) with the three rotary file systems used, either with or without the use of a PG rotary file for glide-path preparation in root canal instrumentation. Apical dentin is considered the danger zone, as the amount of dentin removed is directly proportional to the occlusal forces the tooth receives and withstands in preventing vertical root fracture [23]. The findings of our study also corroborate those of Hartmann et al. [24], which reported that glide-path preparation using engine-driven glide-path files is linked to reduced apical canal transportation and also maintains optimal canal-centering ability.

The limitation of our study was that, despite our sincere attempts to standardize all specimens using strict inclusion criteria, extracted teeth still cannot be completely standardized in terms of canal shape, size, Knoop hardness of root canal dentin, and apical diameter of the root canal.

## Conclusions

Under the parameters of our study, it was found that all three rotary file systems (PTU, PTN, and PTG) showed some deviation from the original root canal geometry or canal path, and also caused canal transportation to a certain extent at all three levels of the root canal (3 mm, 5 mm, 7 mm) during instrumentation. However, the use of the PG rotary file for initial glide-path preparation, followed by the use of PTN rotary files, showed a maximum canal centering ratio at all three levels of the root canal compared to the PTU and PTG rotary file systems used either with or without glide-path.

The use of the PG/PTN rotary file system showed the least canal transportation at the 3 mm level of root canals, whereas at the 5 mm and 7 mm levels, the PG/PTU rotary file system showed the least canal transportation compared to the other file systems used. Further studies are needed to extrapolate the findings of our study to clinical situations.
